# Repetition Time and Echo Time as Determinants of Quantitative Accuracy in Brain Magnetic Resonance Spectroscopy (MRS): A Comprehensive Review

**DOI:** 10.7759/cureus.107517

**Published:** 2026-04-22

**Authors:** Sumit Tyagi, Ashok Sharma, Ashish Kumar Shukla, Subhash Chand Sylonia, Lalit Gupta

**Affiliations:** 1 Department of Radiodiagnosis and Imaging Technology, Santosh Deemed to be University, Ghaziabad, IND; 2 Department of Radiodiagnosis, Saraswathi Institute of Medical Sciences, Hapur, IND

**Keywords:** brain spectroscopy, echo time, magnetic resonance spectroscopy, quantitative accuracy, repetition time

## Abstract

Magnetic resonance spectroscopy (MRS) is a non-invasive technique that can be used to characterize and measure in vivo biochemical metabolites in the human brain. MRS extends the concept of conventional magnetic resonance imaging, which primarily depicts anatomical structures, to provide metabolic data vital for comprehending the physiological functioning of a normal brain as well as pathological processes such as tumors, neurodegenerative disorders, epilepsy, metabolic encephalopathies, and psychiatric illnesses. To support clinical diagnosis and metabolite research, accurate quantification of brain metabolites, such as N-acetylaspartate, choline-containing substances, creatine, myo-inositol, glutamate, glutamine, and lactate, is vital.

## Introduction and background

Magnetic resonance spectroscopy (MRS) is a non-invasive technique that can be used to characterize and measure in vivo biochemical metabolites in the human brain. MRS extends the concept of conventional magnetic resonance imaging, which primarily depicts anatomical structures, to provide metabolic data vital for comprehending the physiological functioning of a normal brain as well as pathological processes such as tumors, neurodegenerative disorders, epilepsy, metabolic encephalopathies, and psychiatric illnesses [1]. To support clinical diagnosis and metabolite research, accurate quantification of brain metabolites, such as N-acetylaspartate, choline-containing substances, creatine, myo-inositol, glutamate, glutamine, and lactate, is vital.

Nevertheless, the quantitative reliability of MRS depends on a number of acquisition parameters, among which repetition time (TR) and echo time (TE) are the most critical [2]. TR is defined as the time between successive sets of radiofrequency excitation pulses. TE is the time delay between the pulse sequence that prepares for MRS measurement and subsequent signal acquisition; transverse relaxation and signal attenuation occur during this time. The impact of these two parameters is wide-ranging, affecting signal intensity, relaxation weighting, spectral quality, reproducibility, and metabolite detectability [3].

In proton brain MRS, TR controls the degree of longitudinal relaxation recovery, and TE controls the degree of transverse relaxation and J-coupling effects. Therefore, an improper choice of TR and TE significantly impacts quantitative accuracy, causing substantial bias in relative and absolute metabolite signals and resulting in errors in quantification [4].

Short TRs may result in incomplete longitudinal relaxation and thereby provide different T1 saturation effects to metabolites that have different longitudinal relaxation times. This phenomenon is pronounced for metabolites whose T1 values depend on tissue type, pathological condition, and the strength of the magnetic field [5].

Moreover, T1 saturation may cause underestimation of metabolite concentrations, especially in quantitative or multicenter studies. Acquisitions with a long TR reduce T1 weighting and enhance quantitative accuracy but require a longer scan time, which could restrict clinical applicability and increase susceptibility to motion artifacts [6].

TE is also important, as it determines signal decay due to T2 relaxation and governs spectral complexity. Acquisitions that use a short TE preserve the signal of metabolites that have a small T2 and thereby enable observation of those metabolites, including short T2 spectral peaks for myo-inositol and glutamate-glutamine complexes [7].

However, short TE spectra commonly exhibit many overlapping peaks and baseline distortion due to macromolecules and lipids, which makes spectral fitting and quantification more difficult. Systematic errors may arise from macromolecular signals unless they are modeled correctly. In contrast, long TE acquisitions produce more easily interpreted spectral peaks, but selectively attenuate or even eliminate spectral peaks for metabolites with shorter T2, which may result in an incomplete representation of the metabolite profile and a skewed estimate of concentration [8].

The interaction between TR and TE is of special concern for quantitative MRS techniques, such as absolute concentration measurements, metabolite ratios, and longitudinal or multicenter studies [9]. The fact that different scanners and institutions use different acquisition protocols further complicates the attainment of comparable and reproducible results, increasing inter-site variability. Because MRS is becoming less of a qualitative adjunct and more of a quantitative biomarker in neuroscience and clinical research, the effects of TR and TE on the quantitative accuracy of brain MRS need to be carefully evaluated. Therefore, standardization and optimization of these parameters are essential to improve the reliability of MRS-based metabolic biomarkers in both clinical practice and research [10].

## Review

MRS has progressed to become a useful tool for assessing brain metabolism for both clinical and research purposes. In contrast to conventional magnetic resonance imaging, which primarily depicts anatomical structures, MRS enables measurement of biochemical metabolites that indicate neuronal integrity, membrane turnover, energy metabolism, and cellular proliferation [11]. These quantitative measurements can be highly inaccurate depending on the acquisition parameters, among which TR and TE are two of the key determinants. The impact of these two parameters is wide-ranging, affecting signal intensity, relaxation effects, spectral quality, and reproducibility, and they are of prime consideration in protocol design.

In a complementary age-related metabolite study, Yang et al. [12] performed proton MRS across a wide age range using a single TR/TE protocol (TR = 2,000 ms, TE = 30 ms) to measure absolute metabolite concentrations in multiple brain regions. Their results demonstrated age-related changes in N-acetylaspartate and myo-inositol, but importantly, they attributed variability in previously published age effects partly to differences in acquisition parameters such as TR and TE among studies. This underscores how protocol inconsistency can confound biological interpretations and how the selection of TR and TE must be carefully considered when comparing metabolite levels across populations or conditions.

TR controls the degree of longitudinal relaxation recovery of metabolites between repeated excitations [12]. Different metabolites measured in brain MRS, such as N-acetylaspartate, choline-containing compounds, and creatine, have varying T1 relaxation times that depend on tissue composition, strength of the magnetic field, and pathological condition. When TR is short compared to a metabolite's T1 value, incomplete relaxation will produce signal saturation that will lead to underestimation of that metabolite's concentration [13].

Rizzo and Kreis [13] directly focused on the influence of TE on quantitative MRS analysis. Their work at 3 T evaluated metabolite quantification at TE values of 72, 144, and 288 ms and compared results using LCModel (see lcmodel.com) via T2-corrected reference methods. They found that longer TE values can lead to overestimation of metabolite levels when fitting algorithms do not fully account for T2 decay, and that this overestimation varies by metabolite and pathology. This highlights a critical point: even though a longer TE may reduce baseline complexity, failure to correct for T2 decay therefore results in reduced reliability of quantification, especially when software assumes simplified signal decay. Therefore, TE selection and the post-acquisition model used both contribute to quantitative uncertainty.

Acquisitions with short TRs have been used to shorten scan duration, especially in clinical settings. However, there is a trade-off in quantitative accuracy, because T1 weighting has an uneven impact on metabolites with longer relaxation times. Research has shown that protocols that use short TRs can generate variability in metabolite ratios, in particular the ratio of N-acetylaspartate to creatine, which can be confusing to interpret in longitudinal or comparative research studies. Conversely, acquisitions with a long TR permit nearly complete relaxation between acquisitions, which reduces saturation effects and enhances absolute quantification reliability [14]. Table 1 summarizes a comparison between short and long TR protocols and their effect on signal accuracy.

**Table 1:** Effect of repetition time (TR) on metabolite quantification accuracy in brain MRS Synthesized from Yang et al. [12] MRS: magnetic resonance spectroscopy

TR category	Degree of T1 recovery	Impact on metabolite signal	Effect on quantification	Clinical implication
Short TR (<1,500 ms)	Incomplete	Variable T1 saturation across metabolites	Underestimation (T1 bias)	Suitable for rapid screening only
Intermediate TR (1,500-2,000 ms)	Partial to near-complete	Moderate variability	Acceptable with T1 relaxation correction (e.g., relaxation modeling or internal water referencing)	Compromise between scan time and accuracy
Long TR (>2,000 ms)	Near-complete	Stable signal amplitudes	High quantitative accuracy	Preferred for research and longitudinal/multicenter studies

The level of transverse relaxation and signal attenuation prior to acquisition is determined by TE. In particular, metabolites with short T2 values are highly sensitive to the selection of TE. Short TE acquisitions can be used to detect a broad range of metabolites, such as myo-inositol, glutamate, glutamine, and macromolecules, which makes short TE protocols effective for full metabolic profiling [15]. However, short TE spectra exhibit a complicated baseline and overlapping peaks, which makes spectral fitting and quantification more difficult. Systematic errors may exist because of macromolecular signals, unless they are correctly modeled. In contrast, long TE acquisitions produce more easily interpreted spectral peaks, but selectively attenuate peaks with shorter T2, which can result in an incomplete representation of the metabolite profile [16]. Table 2 explains the advantages and disadvantages of different TE choices.

**Table 2:** Influence of echo time on metabolite detectability and spectral reliability Synthesized from Viallon et al. [16]

Metabolite	T1 relaxation time	Factors affecting T1 relaxation
N-Acetylaspartate (NAA)	700-1,500 ms	Tissue composition (gray/white matter), magnetic field strength, neurodegenerative conditions (e.g., Alzheimer's)
Choline-containing compounds (e.g., choline and phosphocholine)	700-1,500 ms	Tissue composition, cell membrane integrity, magnetic field strength, pathological conditions (e.g., tumors and gliomas)
Creatine (Cr)	200-400 ms	Magnetic field strength, tissue composition, metabolic demand, brain vs. muscle tissue
Lactate	200-400 ms	Pathological conditions (e.g., ischemia and hypoxia), tissue type, magnetic field strength
Glutamate/glutamine (Glx)	500-1,000 ms	Tissue composition, neurological disorders (e.g., epilepsy and neurodegeneration), magnetic field strength

The interaction between TR and TE is significant for quantitative accuracy overall. For example, a short TR combined with a long TE results in compounded signal loss due to both T1 saturation and T2 decay. In contrast, a long TR and a short TE maximize signal recovery but require a longer scan time and more complex post-processing [17]. Quantitative MRS techniques that depend on absolute concentration measurement, for example, by the use of water as a reference or an external phantom, are particularly vulnerable to these combined effects. Differences in TR and TE among scanners and institutions are additional factors that cause inter-site variability and constrain the comparability of studies. Table 3 summarizes typical TR and TE combinations used in brain MRS and their quantitative effects [18].

**Table 3:** Combined impact of repetition time and echo time on quantitative reliability Synthesized from Yamamoto et al. [19]

Echo time (TE) category	T2 relaxation effect	Metabolites detected	Baseline/macromolecule contribution	Quantitative limitation
Short TE (20-35 ms)	Minimal decay	N-Acetylaspartate, choline, creatine, myo-inositol, glutamate	High macromolecule and lipid contribution	Complex spectral fitting required
Intermediate TE (~135 ms)	Moderate decay	N-Acetylaspartate, choline, creatine, lactate	Reduced baseline interference	Partial loss of short T2 metabolites
Long TE (>200 ms)	Significant decay	N-Acetylaspartate, choline	Minimal macromolecule contribution	Loss of metabolite diversity and sensitivity

In clinical practice, MRS is frequently used for lesion characterization, tumor grading, and differentiation of recurrent tumors from radiation injury. In such applications, metabolite ratios are often preferred to metabolite quantification, which may partially mitigate the effects of TR and TE variability [20]. However, even ratio-based analyses can be biased if relaxation effects differ substantially between metabolites. In research settings, particularly in longitudinal and multicenter studies, absolute quantification is increasingly preferred, which necessitates rigorous control of acquisition parameters. Standardization efforts have emphasized the need for harmonized TR and TE protocols to improve reproducibility and data sharing. Table 4 presents recommended parameter values for different clinical and research objectives [19].

**Table 4:** Recommended repetition time (TR) and echo time (TE) selection based on study objective Adapted clinical protocol trends reported by Yamamoto et al. [19] NAA: N-acetylaspartate

Application	Goal	Recommended TR	Recommended TE	Rationale
Routine clinical diagnosis	Ratio-based analysis	Intermediate	Intermediate-long	Faster acquisition with cleaner spectra
Brain tumor evaluation	Choline/NAA assessment	Long	Intermediate (~135 ms)	Improved contrast and lactate visibility
Neurochemical research	Absolute quantification	Long	Short	Maximizes metabolite detection and accuracy
Multicenter studies	Harmonization	Standardized long TR	Fixed TE	Reduces inter-site variability

Table 5 shows that TR has a direct effect on signal recovery, quantification, and acquisition efficiency in brain MRS. Short TR values lead to incomplete longitudinal recovery and greater T1 weighting, which shortens scan time but reduces quantitative accuracy, whereas longer TR values allow more complete recovery of metabolite magnetization and therefore improve quantitative reliability, albeit at the cost of a longer examination time. For this reason, shorter TRs are generally used when speed is prioritized, while longer TRs are preferred for research protocols and more robust metabolite quantification.

**Table 5:** Influence of TR selection on brain MRS signal characteristics Synthesized from Knight-Scott et al. [21] TR: repetition time; MRS: magnetic resonance spectroscopy

TR range	Longitudinal recovery	Quantitative accuracy	Scan time	Typical application
Short (<1,500 ms)	Incomplete	Lower	Short	Rapid clinical screening
Intermediate (1,500-2,000 ms)	Partial to near-complete	Moderate	Moderate	Routine clinical MRS
Long (>2,000 ms)	Near-complete	Higher	Long	Research studies and absolute quantification

Table 6 compares the practical consequences of using short and long TE acquisitions in brain MRS. Short TE spectra provide broader metabolite coverage because more spectral peaks are retained, but they are also more complex and contain stronger macromolecular and lipid contributions, which makes interpretation and quantification more difficult. In contrast, long TE spectra produce more easily interpreted spectral peaks, because many short T2 and macromolecular signals are attenuated, although this simplification comes at the cost of reduced visibility for several metabolites. Clinically, this is why short TE is often favored for metabolic profiling, whereas longer or intermediate TE is frequently used when a cleaner spectrum is needed, including in tumor assessment.

**Table 6:** Comparison of short and long TE brain MRS Synthesized from Wilson et al. [22] TE: echo time; MRS: magnetic resonance spectroscopy

Parameter	Short TE	Long TE
Metabolite coverage	Broad	Limited
Spectral complexity	High	Low
Macromolecule contribution	Prominent	Suppressed
Quantification difficulty	Higher	Lower
Common use	Metabolic profiling, research	Tumor evaluation, routine clinical use

Table 7 illustrates that the effect of TE is metabolite-specific. N-Acetylaspartate, choline, and creatine remain reliably visible at both short and long TE, which explains their central role in routine clinical interpretation. In contrast, myo-inositol is much better appreciated at short TE because its signal is relatively vulnerable to T2 decay and spectral simplification at longer TE. Lactate behaves differently, because although it may be detectable at short TE, it is often more conspicuous at intermediate or long TE, especially around 135-144 ms, where J-coupling causes its doublet spectral peak to invert and become easier to distinguish from overlapping lipid resonances.

**Table 7:** Effect of TE on selected brain metabolites Synthesized from Wilson et al. [22] TE: echo time

Metabolite	Short TE	Long TE	Clinical relevance
N-Acetylaspartate	High	High	Neuronal integrity
Choline	High	High	Membrane turnover
Creatine	High	High	Energy metabolism
Myo-inositol	High	Low	Gliosis
Lactate	Moderate	High (TE ~135-144 ms, J-coupling inversion)	Hypoxia, tumors

Table 8 summarizes commonly used TR and TE combinations for major brain MRS applications. In brain tumors, intermediate or long TE values around 135-144 ms are often selected when lactate detection and spectral simplification are important, although short TE protocols are also widely used when broader metabolic characterization is required. For metabolic disorders, neurodegenerative conditions, and many pediatric studies, a long TR and a short TE are generally preferred because this combination maximizes metabolite detection and preserves compounds such as myo-inositol and other short T2 resonances. These protocol choices should therefore be understood as practical recommendations rather than rigid standards, since the optimal combination still depends on field strength, sequence design, and the diagnostic question.

**Table 8:** Comparative synthesis of key studies evaluating TR and TE in brain MRS Synthesized from Wijtenburg and Knight-Scott [23] TE: echo time; TR: repetition time; MRS: magnetic resonance spectroscopy

Clinical application	Preferred TR	Preferred TE
Brain tumors	≥1,500 ms	135-144 ms
Metabolic disorders	≥2,000 ms	20-35 ms
Neurodegeneration	≥2,000 ms	20-35 ms
Pediatric studies	1,500-2,000 ms	Short TE

This review places the influence of TR and TE on quantitative accuracy in brain MRS in context with four key prior investigations. Across these studies, the consistent theme is that both TR and TE systematically influence metabolite signal amplitudes, relaxation effects, and the reliability of both absolute and ratio-based metabolite quantification.

Knight-Scott et al. [21] investigated the impact of TR on absolute and relative metabolite concentrations in human brain proton MRS at 3 T. They acquired spectra at multiple TR values and demonstrated that short TR values produce significant deviations in metabolite ratios due to T1 weighting, and concluded that a minimum TR of approximately 2.5 s is recommended for ratio-based measurements and approximately 5 s for absolute quantification. Their analysis found that the variance in absolute concentrations was smaller than that of ratios, indicating that a long TR improves quantitative accuracy by minimizing T1 saturation effects. This study directly supports the hypothesis that standardizing TR is essential for reliable quantification, especially in longitudinal or multicenter studies.

In a related methodological consensus report, Wilson et al. [22] emphasized the need for optimized acquisition and analysis protocols to improve MRS reliability and reproducibility across clinical and research settings. While not focused exclusively on TR or TE, this consensus highlighted that short TE acquisitions provide broader metabolite visibility but require advanced spectral basis sets for analysis, and that sequences with improved localization, such as semi-LASER, help reduce methodological variance across studies. Their recommendations also affirm that a consistent choice of TR and TE is a prerequisite for harmonized quantitative assessments in multicenter studies, as demonstrated in Table 9.

**Table 9:** Critical synthesis of major studies evaluating repetition time (TR) and echo time (TE) in brain magnetic resonance spectroscopy (MRS), highlighting methodological approaches, strengths, limitations, and their contribution to improving quantitative reliability

Author and reference	Study design & methodology	Key findings on TR/TE	Strengths	Limitations	Critical contribution to the field
Knight-Scott et al. [21]	Multi-TR experimental study at 3 T evaluating metabolite ratios and absolute quantification	Short TR introduces T1 bias; ≥2.5 s improves ratios; ≥5 s improves absolute quantification	Direct experimental comparison of TR values	Limited generalizability across brain regions and pathologies	Established TR thresholds for reliable quantification and highlighted T1 weighting bias
Yang et al. [12]	Cross-sectional population study using fixed TR/TE protocol	Demonstrated age-related metabolite variation; highlighted parameter-dependent variability across studies	Large population-based dataset	Did not vary TR/TE → cannot isolate acquisition effects	Showed that biological conclusions may be confounded by protocol differences
Yamamoto et al. [19]	Comparative TE analysis (72-288 ms) with LCModel and T2 correction	Long TE simplifies spectra but causes metabolite-specific overestimation if T2 not corrected	Direct comparison of TE and modeling approaches	Strong dependence on post-processing assumptions	Demonstrated that TE-related errors are both acquisition- and model-dependent
Wilson et al. [22]	Consensus guideline and methodological review	Advocates standardized TR/TE and advanced localization (semi-LASER)	Multicenter perspective and standardization framework	Limited empirical validation across all scanners	Established need for harmonization in clinical and research MRS
Wijtenburg and Knight-Scott [23]	Technical study on very short TE acquisition at 3 T	Short TE improves the detection of glutamate and complex metabolites	Improved spectral sensitivity	Increased spectral overlap and complexity	Reinforced role of short TE in advanced metabolic profiling

## Conclusions

TR and TE are critical determinants of quantitative accuracy in brain MRS, as they directly influence T1 and T2 relaxation effects, signal intensity, and spectral quality. Short TR values introduce metabolite-specific T1 saturation, leading to underestimation of concentrations, whereas longer TR values (≥2,000-2,500 ms) allow near-complete longitudinal recovery and improve quantitative reliability. Similarly, TE selection involves a trade-off: short TE enhances detection of a wider range of metabolites, including short T2 compounds such as myo-inositol and glutamate, but increases spectral complexity, while longer TE simplifies spectra at the cost of signal loss and reduced metabolite diversity.

Importantly, the interaction between TR and TE has a compounded effect on signal behavior, with long TR and short TE providing the most robust conditions for accurate quantification, albeit with increased acquisition time and processing requirements. Variability in TR and TE across studies and imaging centers remains a significant source of inter-site inconsistency, particularly in multicenter and longitudinal research. Therefore, careful selection and standardization of TR and TE, along with appropriate relaxation correction and advanced spectral modeling techniques, are essential to improve reproducibility, comparability, and consistency of results across different studies and imaging centers.

## References

[1] Veeraiah P, Jansen JF (2023). Multinuclear magnetic resonance spectroscopy at ultra-high-field: assessing human cerebral metabolism in healthy and diseased states. Metabolites.

[2] Weinberg BD, Kuruva M, Shim H, Mullins ME (2021). Clinical applications of magnetic resonance spectroscopy in brain tumors: from diagnosis to treatment. Radiol Clin North Am.

[3] Zhao G, Zhang H, Xu Y, Chu X (2024). Research on magnetic resonance imaging in diagnosis of Alzheimer's disease. Eur J Med Res.

[4] Tyagi S, Sharma A, Sylonia SC, Gupta L (2025). Role of TR (repetition time) and TE (echo time) in optimization of magnetic resonance spectroscopy in the brain protocol. Natl J Med Res.

[5] Alwahsh M, Nimer RM, Dahabiyeh LA, Hamadneh L, Hasan A, Alejel R, Hergenröder R (2024). NMR-based metabolomics identification of potential serum biomarkers of disease progression in patients with multiple sclerosis. Sci Rep.

[6] Wright AM, Murali-Manohar S, Henning A (2022). Quantitative T(1)-relaxation corrected metabolite mapping of 12 metabolites in the human brain at 9.4 T. Neuroimage.

[7] Carlin D, Babourina-Brooks B, Davies NP, Wilson M, Peet AC (2019). Variation of T(2) relaxation times in pediatric brain tumors and their effect on metabolite quantification. J Magn Reson Imaging.

[8] Hong S, Shen J (2023). Neurochemical correlations in short echo time proton magnetic resonance spectroscopy. NMR Biomed.

[9] Hagiwara A, Fujita S, Ohno Y, Aoki S (2020). Variability and standardization of quantitative imaging: monoparametric to multiparametric quantification, radiomics, and artificial intelligence. Invest Radiol.

[10] Shan D, Zhang Y, Wang M, Liu Y, Wang Y, Xiao L (2025). Value of magnetic resonance spectroscopy for examining fetal brain development in mid- to late pregnancy. iRADIOLOGY.

[11] Krauss W, Gunnarsson M, Andersson T, Thunberg P (2015). Accuracy and reproducibility of a quantitative magnetic resonance imaging method for concurrent measurements of tissue relaxation times and proton density. Magn Reson Imaging.

[12] Yang ZY, Yue Q, Xing HY (2015). A quantitative analysis of (1)H-MR spectroscopy at 3.0 T of three brain regions from childhood to middle age. Br J Radiol.

[13] Rizzo R, Kreis R (2023). Multi-echo single-shot spectroscopy combined with simultaneous 2D model fitting for fast and accurate measurement of metabolite-specific concentrations and T(2) relaxation times. NMR Biomed.

[14] Marjańska M, Emir UE, Deelchand DK, Terpstra M (2013). Faster metabolite (1)H transverse relaxation in the elder human brain. PLoS One.

[15] Dvorak AV, Ljungberg E, Vavasour IM (2021). Comparison of multi echo T(2) relaxation and steady state approaches for myelin imaging in the central nervous system. Sci Rep.

[16] Viallon M, Leporq B, Drinda S, Wilhelmi de Toledo F, Galusca B, Ratiney H, Croisille P (2019). Chemical-shift-encoded magnetic resonance imaging and spectroscopy to reveal immediate and long-term multi-organs composition changes of a 14-days periodic fasting intervention: a technological and case report. Front Nutr.

[17] Kreis R, Boer V, Choi IY (2020). Terminology and concepts for the characterization of in vivo MR spectroscopy methods and MR spectra: background and experts' consensus recommendations. NMR Biomed.

[18] Tolia M, Verganelakis D, Tsoukalas N (2015). Prognostic value of MRS metabolites in postoperative irradiated high grade gliomas. Biomed Res Int.

[19] Yamamoto T, Isobe T, Akutsu H (2015). Influence of echo time in quantitative proton MR spectroscopy using LCModel. Magn Reson Imaging.

[20] Ho BC, Kim D, Kumar A, Weiss S, Vossler H, Mormino E, Zaharchuk G (2026). Evaluation of image-level harmonization methods for multi-center MR neuroimaging. J Magn Reson Imaging.

[21] Knight-Scott J, Brennan P, Palasis S, Zhong X (2017). Effect of repetition time on metabolite quantification in the human brain in (1) H MR spectroscopy at 3 Tesla. J Magn Reson Imaging.

[22] Wilson M, Andronesi O, Barker PB (2019). Methodological consensus on clinical proton MRS of the brain: review and recommendations. Magn Reson Med.

[23] Wijtenburg SA, Knight-Scott J (2011). Very short echo time improves the precision of glutamate detection at 3T in 1H magnetic resonance spectroscopy. J Magn Reson Imaging.

